# Unraveling the mechanisms of bone diseases: targeting dendritic cells in osteoimmunology for internal homeostasis balance

**DOI:** 10.1038/s41413-025-00456-7

**Published:** 2025-09-28

**Authors:** Yanqi Chen, Siyuan Wang, Xiaoyu Chen, Zhifang Wu, Fuming He, Qianming Chen

**Affiliations:** 1 Department of Pediatrics, Stomatology Hospital, School of Stomatology, Zhejiang University School of Medicine, Zhejiang Province Clinical Research Center for Oral Disease, Key Laboratory of Oral Biomedical Research of Zhejiang Province, Cancer Center of Zhejiang University, Engineering Research Center of Oral Biomaterials and Devices of Zhejiang Province, Hangzhou, China; 2 Department of Prosthodontics, Stomatology Hospital, School of Stomatology, Zhejiang University School of Medicine, Zhejiang Province Clinical Research Center for Oral Disease, Key Laboratory of Oral Biomedical Research of Zhejiang Province, Cancer Center of Zhejiang University, Engineering Research Center of Oral Biomaterials and Devices of Zhejiang Province, Hangzhou, China; 3 Stomatology Hospital, School of Stomatology, Zhejiang University School of Medicine, Zhejiang Province Clinical Research Center for Oral Disease, Key Laboratory of Oral Biomedical Research of Zhejiang Province, Cancer Center of Zhejiang University, Engineering Research Center of Oral Biomaterials and Devices of Zhejiang Province, Hangzhou, China

**Keywords:** Bone, Pathogenesis

## Abstract

Bone repair and regeneration is a complex spatiotemporal process recruiting a variety of cell types, which need to precisely mediated for effective healing post-damage. The concept of osteoimmunology emphasizes the extensive and intricate crosstalk between the bone and the immune system. Despite the significant advancements in understanding osteoimmunology, the precise role of dendritic cells (DCs) in this field remains under investigation. As key antigen-presenting cells, DCs are critical in orchestrating adaptive immune responses and maintaining tissue homeostasis. Recent researches have further revealed the potential of DCs to influence the development or acceleration of inflammatory and autoimmune bone disease, as well as their interaction with skeletal cells in the context of bone repair and regeneration. Therefore, an in-depth understanding of DCs in the osteoimmunology would be valuable. Herein, we discuss the effects of DCs on bone homeostasis and bone-related diseases (i.e., rheumatoid arthritis (RA), periodontitis, bone regeneration, and other bone abnormalities diseases), and introduce the innovative DCs-targeting biomaterials, aimed at promoting bone repair and regeneration. Furthermore, we summarize the underlying crosstalk between DCs and other cells (i.e., osteoclasts, mesenchymal stromal stem cells (MSCs), hematopoietic stem and progenitor cells (HSPCs), T and B cells) in the bone homeostasis and bone-related diseases. In conclusion, we propose that osteoimmunology offers a promising perspective for unraveling the mechanisms of bone-related diseases; meanwhile, targeting DCs from the perspective of osteoimmunology may provide innovative ideas and resolutions to achieve the internal homeostasis balance.

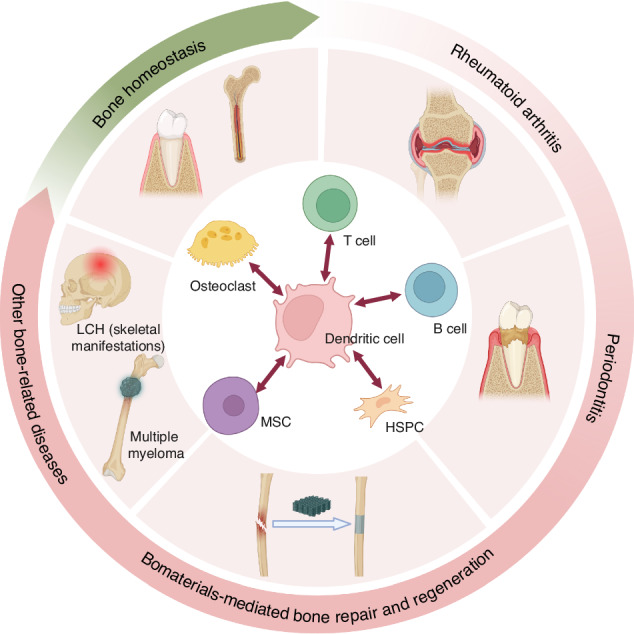

## Introduction

Bone diseases are common clinical conditions that can severely impair function and diminish the quality of life.^1,2^ Despite the rapid advancements in tissue engineering, clinicians still grapple with the challenges posed by bone defects, particularly large-area injuries.^3^ Recently, many studies shed light on complex interactions between the bone and immune systems. Immune cells (i.e., neutrophils, monocytes (Mo), macrophages (Mφ), dendritic cells (DCs), T and B cells) are involved in the balance between the processes of bone formation and bone resorption linked to various bone diseases, such as rheumatoid arthritis (RA), periodontitis, delayed bone regeneration, and other bone abnormalities diseases.^4,5^ Cytokine production (i.e., RANKL, GM-CSF, TGF-β) during immune responses could profoundly affect bone homeostasis by altering the balance between bone-forming osteoblasts (OBs) and bone-resorbing osteoclasts (OCs). For instance, regulatory T cells (Treg) inhibited osteoclastogenesis by producing suppressor cytokines, including IL-4, IL-10, and TGF-β, whereas IL-17, RANKL, TNF-α, IL-1, and IL-6 secreted by Th17 cells exerted osteoclastogenic activity.^6^ On the other hand, the immune system is spawned in the bone marrow reservoir, where important niches existed for immune cell formation and interaction with bone cells.^7^ Therefore, cells from the immune and bone systems share the same microenvironment and interact extensively.

Based on the observation of bone growth disruption and restructuration in autoimmune diseases, Arron and Choi put forward the term ‘osteoimmunology’ in 2000 first to delineate the intricate regulatory interactions between the bone and immune system.^8^ With the advancements in studies on the crosstalk between the two systems and our cognition, the conceptual framework of osteoimmunology has been further updated.^9,10^ DCs, as potent antigen-presenting cells responsible for the activation of quiescent T cells and the orchestration of the immune response, have emerged as a critical component of the bone-immune interface.^11,12^ In bone homeostasis, recent study has confirmed that a majority of DCs reside perivascularly in the bone marrow, where they play a critical role in the trafficking of hematopoietic stem and progenitor cells (HSPCs), thereby contributing to the regulation of hematopoiesis and the establishment of the stem cell niche.^13^ Conversely, in pathological conditions such as chronic periodontitis, DCs are proposed to indirectly influence inflammatory bone resorption by activating and regulating T cell function.^14^ In addition, the heterogeneity and diversity of DCs subsets significantly contribute to the pathogenesis of periodontitis.^15^ Beyond the interactions between DCs and T cells, the interactions between DCs and B cells, as well as the feedback loop involving DCs and OCs, also lead to severe bone destruction and play a role in inflammatory bone resorption, as observed in RA.^16^ Interestingly, increased studies have revealed that biomaterials can influence the polarization of DCs through their intrinsic properties,^17,18^ thereby modulating the local immune environment to reverse the progression of inflammatory bone-related diseases^19,20^ and enhance biomaterials-mediated bone repair and regenertion.^21,22^ These findings suggest that DCs are key players in the field of osteoimmunology.

Herein, we examine the current understanding of DCs in the osteoimmunology, focusing on their distribution, heterogeneity and functional changes associated with the occurrence and progression of bone-related diseases, such as RA, periodontitis and other bone abnormalities diseases. We also explore their role in maintaining bone homeostasis. Additionally, we summarize the underlying crosstalk between DCs and various cell types, including OCs, OBs, HSPCs, T and B cells within the bone microenvironment. Furthermore, we introduce novel strategies concerning the role of DCs in bone-related diseases, including DCs-targeted vaccines, epigenetic reprogramming, and DCs-targeted biomaterials aimed at promoting bone repair and regeneration. We anticipate that this review will provide valuable insights into potential therapeutic targets for achieving a balance in bone homeostasis, serving as a cornerstone for the future development of treatment modalities.

## Overview of DCs

### DCs serve as the bridge between innate and adaptive immunity

As early as the mid 1970s, Prof. Ralph Steinma characterized and nominated a particular subgroup with ‘dendritic’ morphology in the spleens with DCs as effective stimulator of innate immune response.^23^ At that time, most immunologists speculated that Mφ instead of DCs, served as the most potent antigen-presenting cells (APCs) with plentiful populations and even distribution.^24^ The potent antigen-presenting effects of bone marrow-resident DCs (BMDCs) have not been clarified until the 80 years last century when researchers reported that DCs can activate T cells in the mixed lymphocyte reactions (MLR) in a manner 100 times more efficient than Mφ.^25,26^

**Fig. 1:**
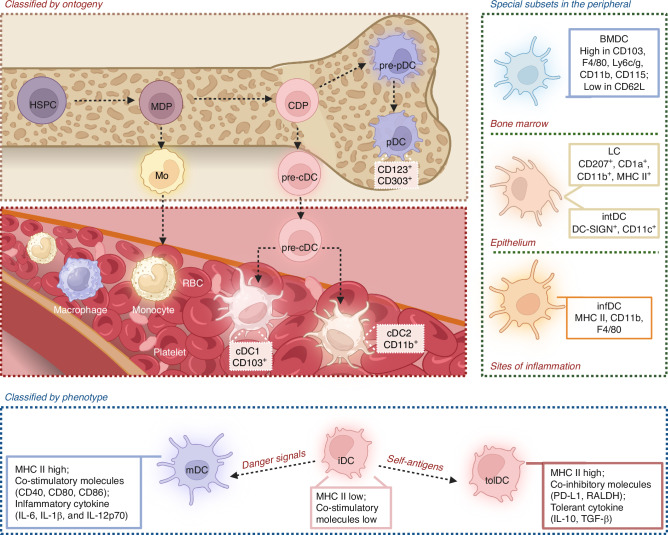
Dendritic cell (DCs) subtypes involved in bone homeostasis and disease. DCs are a highly heterogeneous population of antigen-presenting cells (APCs) that can be classified into distinct subpopulations based on their ontogeny, phenotype, and function. DCs originate from bone marrow myeloid progenitor cells (MDPs) derived from hematopoietic stem and progenitor cells (HSPCs). These progenitors can give rise to common dendritic cell progenitors (CDPs), which further differentiate into two major types: conventional DCs progenitors (pre-cDCs), which develop into cDCs in the periphery, and plasmacytoid DCs progenitors (pre-pDCs), which differentiate into pDCs in the bone marrow. Other subtypes of DCs, including Langerhans cells (LCs) and interstitial DCs (intDCs), are localized in the epithelium, while bone marrow DCs (BMDCs) are found in the bone marrow. Additionally, inflammatory DCs (infDCs) are present at peripheral inflammation sites. Functionally, immature DCs (iDCs) can encounter danger signals, resulting in their maturation into mature DCs (mDCs) and promotion of immune responses. Conversely, when iDCs encounter self-antigens without sufficient co-stimulatory signals, they differentiate into tolerogenic DCs (tolDCs) and induce immunomodulation. Mo monocyte, RBC red blood cell

DCs derived from bone marrow myeloid progenitor cells from HSPCs in a Flt3L-dependent manner^27,28^ (Fig. 1). Common dendritic cell progenitors (CDPs) can be divided into conventional DC progenitors (pre-cDCs) and plasmacytoid DC progenitors (pre-pDCs), which develop into cDCs in the periphery and pDCs in the bone marrow, respectively.^27,28^ Subsequently, DCs served as sentinel in the immune microenvironment, comprising the first defensive line in the immune system. DCs can recognize pathogen-associated molecular patterns (PAMPs) and damage-associated molecular patterns (DAMPs) via membrane specific pathogen recognizing receptors (PRRs), induce the production of cytokines, and active antigen-specific T cells migrating to the lymph nodes, which are therefore acknowledged as the most potent APCs.^29,30^ After exposure to PAMPs/DAMPs, DCs experienced phenotypic conversion from immature dendritic cells (iDCs) to mature dendritic cells (mDCs) (Fig. 1). Specifically, iDCs possessed relatively low expression levels of co-stimulatory molecules (i.e., CD40, CD83, CD80, and CD86) and major histocompatibility complex (MHC) I/II, with weak capacity to induce T cells. Whenever it matured with a dendritic morphology, mDCs activated T cells and promoted immune responses by higher expression levels of co-stimulatory molecules and MHC I/II.^31^ Nevertheless, when DCs encountered non-antigen (self-antigens), they developed into tolerogenic phenotypes (tolDCs) leading to T cell anergy and/or development of Treg.^32^ TolDCs maintained low expression levels of CD86 and CD80, increased production of IL-10 whilst reduced secretion of IL-6, IL-1β, and IL-12p70. In this context, tolDCs preserved the ability of antigen presentation but failed to activate T cells without second signals.^32^ Serving as the bridge between innate and adaptive immunity, DCs possessed dualized ability of induce immunogenicity or tolerance, has grown progressively more attention as a viable treatment target.^17,32^

Although the current classification of DCs in the bone marrow microenvironment is less elucidated as in the spleen or tumor, more subsets of DCs are being explored by the development of novel high-throughput tools including single‐cell RNA sequencing (scRNA-seq), such as BMDCs,^13,33^ langerhans cells (LCs),^34^ interstitial DCs (intDCs)^35^ and inflammatory DCs (infDCs),^36^ playing an important role in the bone-related diseases (Fig. 1).

### Bone marrow-resident dendritic cells (BMDCs)

BMDCs consisted of a small population of bone marrow cells (1%–2%), which organized into perivascular clusters enveloping sinusoid and venules without direct contacting the vessel walls indicated by two-photon intravital microscopy.^33^ In addition, F4/80^+^ CD11c^chi^ MHC II^+^ BMDCs stimulated allogeneic naive CD4^+^ T cells in a manner more efficient than F4/80^+^ CD11c^-^ MHC II^-^ Mo or F4/80^+^ CD11c^-^ MHC II^+^ Mφ.^33^ Comparative flow cytometry unveiled that BMDCs differed from the bulk of splenic cDCs with higher expression levels of CD103, F4/80, Ly6c/g, CD11b, and CD115 while lower expression levels of CD62L.^33^ Zhang and colleagues performed ScRNA-seq and reported that BMDCs presented CD11b^+^ XCR1^-^ phenotype resembling cDC2, whereas the gene expression patterns for chemokines, chemokine receptors were strikingly different with cDC2, suggesting that BMDCs might represent a distinct DCs population.^13^

Bone marrow has been acknowledged as a primary lymphoid organ accommodating HSPCs and lymphocytes,^33^ and a secondary lymphoid organ providing favorable microenvironment for the priming and restimulation of adaptive immune response.^37^ Recent studies reported that ablation of BMDCs resulted in HSPC mobilization that was greater than ablation of bone marrow macrophages, thereby constituting bone marrow perivascular hematopoietic niche.^13^ In addition, BMDCs induced rapid proliferation of recirculating mature B cells and CD8^+^ central memory T cells.^33,38^ Sapoznikov and colleagues confirmed that perivascular clusters of BMDCs promoted the maintenance of recirculating B cells via macrophage migration–inhibitory factor (MIF), and conditional ablation of BMDCs resulted in specific reduction of mature B cells.^33^ These studies preliminarily confirmed the critical role of BMDCs in maintaining bone homeostasis.

In addition to the critical role in regulating the activity and differentiation of T cells via RANK/RANKL axis and secretion of osteoclastogenesis-related cytokine,^39,40^ BMDCs can also “directly” contributed to inflammation-related bone erosion with the potential of developing into DCs-derived OCs (DDOC) under pathological conditions.^37,41^ In this context, DCs, serving as the critical component of host defensive system against foreign entities, have becoming emerging avenues of osteoimmunology.

## DCs and bone-related diseases

### Periodontitis

**Fig. 2:**
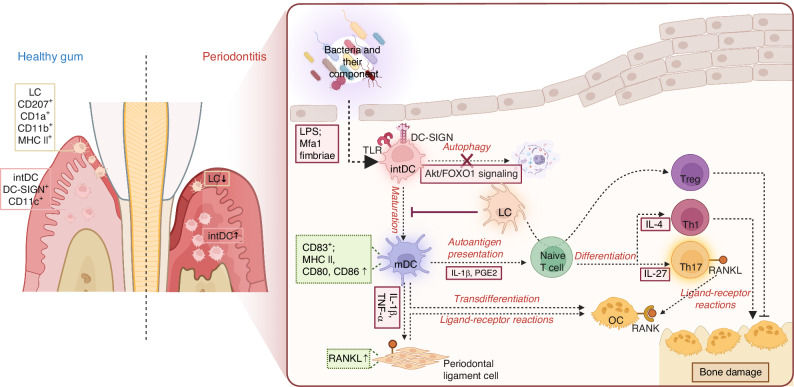
The effects of dendritic cells (DCs) in periodontitis. Periodontitis is a chronic inflammatory disease induced by dysbiosis of the oral microbiota and host immune response, characterized by alterations in the distribution and function of DCs subsets. In the lamina propria, mature interstitial DCs (intDCs) play a central role in driving the immune response and contributing to tissue destruction. These cells mediate antigen presentation to T cells, leading to the activation of effector immune responses that promote bone resorption. In contrast, Langerhans cells (LCs), primarily residing in the gingival epithelium, exert a protective role by inhibiting excessive production of interferon γ (IFN-γ), and increasing the number of regulatory T cells (Tregs). Oral pathogens, such as *Porphyromonas gingivalis* (*P. gingivalis*), utilize lipopolysaccharide (LPS) and Mfa1 fimbriae to interact with DCs, impairing their autophagy and promoting an immunosuppressive environment. Despite this, DCs exposed to these pathogens mature and subsequently induce an effector immune response, contributing to the inflammation and bone damage observed in periodontitis. TLR toll-like receptor, RANK receptor activator of nuclear factor kappa-Β, RANKL receptor activator of nuclear factor kappa-Β ligand

Periodontitis is a chronic inflammatory bone disease induced by dysbiosis of the oral microbiota and host immune response, the latter of which is mainly characterized by dysregulation of T cell and DCs responses^42^ (Fig. 2). DCs aggregate in the lamina propria during chronic periodontitis, mounting T cell-mediated immune response that contributes to periodontal tissue damage.^43^ Thus, DCs are proposed to indirectly affect inflammatory bone resorption through activating and regulating T cell function.^14^

Murine and clinical studies have highlighted that gingival DCs have special localization and function in the pathogenesis of periodontitis.^44,45^ Four distinct subsets of DCs were identified in healthy human gingiva: CD141^+^ cDC1, CD1c^+^ cDC2, CD123^+^ pDCs and CD207^+^ LCs.^45^ CD207^+^ LCs, a distinct subgroup of DCs residing in oral mucosal epithelium, display comparable stimulatory capacity with epidermal LCs.^46^ However, oral mucosal LCs originate from bone marrow-derived precursors, in contrast to epidermal LCs originating from embryonic precursors.^47^ LCs can be identified by the expression of CD11c, MHC II, epithelial cell adhesion molecule (EPCAM), and CD207 in mice^48^ and by the expression of CD207 and CD1a in human.^49^ Based on the expression of CD11c, MHC II, CD11b, CD 103, and CD207, DCs in lamina propria can be divided into four subsets, among which interstitial DCs expressing MHC II, CD 11c and CD11b are found to be the major subset.^48^ More detailed information about DCs subsets in oral mucosa can be found in the review by prof. El-Awady.^50^

Decreased LCs and increased pDCs were observed in the gingiva of periodontitis patients, accompanied by increased expression of proinflammatory cytokines IL-1β, IFN-α, and IFN-γ and suppressed expression of anti-inflammatory cytokines IL-10^45^. Furthermore, smoking was found to specifically reduce the prevalence of LCs in healthy gingiva, indicating a possible role of LCs in the elevated severity of periodontitis in smokers.^45^ LCs also decreased in HIV-seropositive patients with or without chronic periodontitis.^51^ Studies on chronic periodontitis also found that CD207^+^ LCs predominantly infiltrated the gingival epithelium whereas dendritic cell-specific intercellular adhesion molecule-30 grabbing nonintegrin (DC-SIGN/CD209)^+^ interstitial DCs infiltrated the lamina propria, and increasing numbers of CD83^+^ DCs are associated with large clusters of CD4^+^ T cells, indicating that both LCs and interstitial DCs contributed to the mature CD83^+^ DCs population observed in the inflamed gingiva.^52,53^ Actually, the mechanism underlying periodontitis is related to the T cell immune response induced by mature interstitial DCs in the lamina propria, instead of LCs. Biopsies from patients with chronic periodontitis presented that LCs were restricted to the basial layer in pocket epithelium and did not express MHC II antigens nor contact with lymphocytes, while DCs with high expression of MHC II, CD80, and CD86 were abundant in the lamina propria of pocket epithelium, contacting with CD4^+^ and CD8^+^ T cells as well as plasma cells.^43^ It is reported that interstitial DCs mediated antigen presentation to CD4^+^ T cells following *Porphyromonas gingivails (P. gingivalis)* infection, whereas LCs failed to present antigen to CD4^+^ T cells.^35^ Moreover, LCs can inhibit IFN-γ production and excessive activation of RANKL^+^ CD4^+^ T cells through increasing Treg numbers,^35^ while enhanced alveolar bone loss was observed in LCs-ablated mice,^35^ revealing the protective role played by LCs in *P. gingivalis-*induced periodontal bone loss.^34^ Therefore, periodontitis is mainly mediated by mature interstitial DCs in the lamina propria, and LCs play a protective role.

One possible reason for LCs migrating from the diseased situ is associated with the long-term inflammation and the increased commensal load induced by pathogen.^54^ However, there is no definitive evidence for the events happened during the pathological process of periodontitis that resulted in the LCs function inhibition or LCs numbers reduction. Titanium ions released from implants resulted in the generation of LCs precursors (CD11c^+^ MHC II^+^ EPCAM^+^) and the reduction of LCs (CD11c^+^ MHC II^+^ EPCAM^+^ CD207^+^), suggesting that the development of oral LCs could be impaired by titanium dental implants, leading to the peri-implant mucosa immunity dysregulation.^55^ Besides, aging-related changes of LCs have an impact on the severity of periodontal disease.^56^ Reduced gingival LCs with altered morphology were observed in aged mice,^57^ developing an age-related periodontal bone loss.^58,59^ These observations reflect the vital role of LCs in periodontitis and lead to a potential new line of research in the reasons for the decrease of LCs during the pathogenesis of chronic periodontitis.

Furthermore, the adaptive immune response induced by DCs in periodontitis is also influenced by oral pathogens to a large extent. *P. gingivalis*, known as a keystone pathogen in chronic periodontitis,^60^ employs LPS to promote immunosuppression of DCs, inducing weak DCs maturation and a Th2 bias response.^39,61,62^ In vitro studies have shown that *P. gingivalis* and its LPS can not only induce DCs to release pro-inflammatory cytokines involving IL-1β and prostaglandin E2 (PGE2), but also stimulate DCs to secrete anti-inflammatory cytokines such as IL-10 and TGF-β which induce Treg response.^15^ *E. coli* LPS can signal through toll-like receptor 4 (TLR4) and induce DCs maturation and Th1 polarization, whereas *P. gingivalis* LPS depends less on TLR4 and elicit DCs cytokine profile biased to the Th2 response.^61,62^ Moreover, *P. gingivalis* LPS is a potent inducer of matrix metalloproteinase-9 (MMP-9) but a weak inducer of tissue inhibitor of metalloproteinase-1 (an inhibitor of MMP-9) in human DCs, suggesting that DCs pulsed with *P. gingivalis* play a role in local tissue destruction and leukocyte trafficking in gingiva.^63^ Besides, *P. gingivalis* can invade DCs with its minor (Mfa1) fimbriae interacting with C-type lectin receptor DC-SIGN and suppress the maturation of DCs.^64^ The crosstalk between DC-SIGN and TLRs is involved in the mechanisms of *P. gingivalis* with Mfa1 fimbriae evading the intracellular degradation and autophagy. Mfa1 fimbriae have also been shown to perturb DCs autophagy and/or apoptosis through the nuclear/cytoplasmic shuttling of Akt/FOXO1 in DCs, along with upregulated anti-apoptotic protein Bcl-2, decreased caspase-3 and decreased expression of pro-apoptotic protein Bax and Bim.^42^ After immune escape, DCs can harbor and traffic *P. gingivalis* into blood to distant sites,^65^ leading to the development of systemic diseases such as cardiovascular disease, Alzheimer’s disease, and premature low birth weight infants.^66–68^

Based on the above studies, oral DCs and LCs are closely related to periodontitis,^15^ which can be summarized as follows: (1) The special distribution of DCs leaves the gingival tissue vulnerable to the foreign pathogens intrusion; (2) The unique interaction mechanism between periodontitis pathogens and DCs suppress DCs effector immune response and achieve immune escape via DCs; (3) The immune status of DCs in periodontitis are crucial regulator for CD4^+^ T cells differentiation and immune responses, including Th1, Th2, Th17, and Treg^50^; (4) The heterogeneity and diversity of DCs subsets in periodontitis. Except for LCs and interstitial DCs mentioned above, Mo infiltrating the gingival during the inflammation can also transdifferentiate into DCs (known as infDCs), indicating the existence of another DCs subset involved in T cell activation during periodontitis.^54^

DCs are closely related to the occurrence and development of periodontitis due to their critical role in monitoring and reacting to biofilms, giving rise to DCs-based immunotherapy in addition to the traditional treatments for periodontitis.^50,69^ Periodontal vaccines are a proposed approach for the prevention of periodontal disease,^70^ such as DCs-targeted DNA vaccine against FimA which was effective in attenuating periodontitis induced by *P. gingivalis*.^71^ Epigenetic reprogramming of DCs also provides potential therapeutic options.^72^ TolDCs-derived exosomes loaded with TGF-β and IL-10 can reprogram the cellular immune response from Th17 to Treg response and inhibit alveolar bone loss.^19^ Moreover, biomaterials that recruit and program DCs toward a tolerogenic phenotype can enrich Treg cells and hold promise for treating periodontal inflammation.^20^ The strategy of increasing the number of local DCs and inducing DCs tolerance could also be combined with bone regeneration technique in the future to promote bone tissue regeneration while alleviating inflammation.^50^

### Rheumatoid arthritis (RA)

**Fig. 3:**
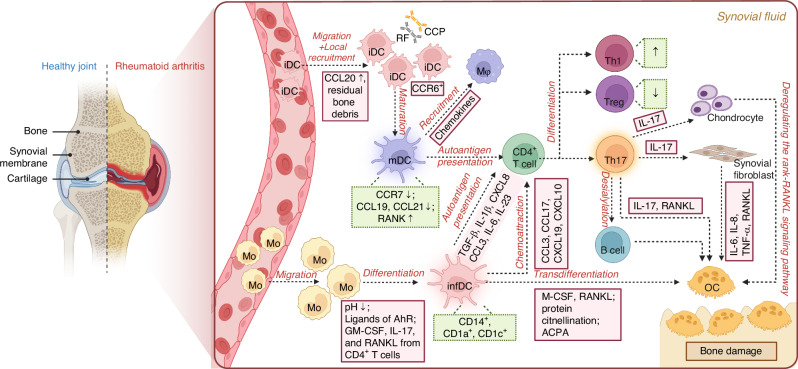
The effects of dendritic cells (DCs) in rheumatoid arthritis (RA). In RA, a chronic autoimmune disease, immature DCs (iDCs) migrate from the blood vessels into the inflamed synovial tissue, where they mature and trigger an effector immune response. These mature DCs (mDCs) activate naive CD4^+^ T cells, which subsequently differentiate into T helper 17 (Th17) cells. Th17 cells release pro-inflammatory cytokines and induce receptor activator of nuclear factor-κB ligand (RANKL) expression in synovial fibroblasts, driving osteoclastogenesis and bone destruction. In addition, monocytes (Mo) that infiltrate the synovium under inflammatory conditions differentiate into inflammatory DCs (infDCs), influenced by low pH, Aryl hydrocarbon receptor (AhR) ligands, and Granulocyte-macrophage colony-stimulating factor (GM-CSF) produced by CD4^+^ T cells. These infDCs further transdifferentiate into osteoclasts (OCs), directly contributing to bone erosion, or promote Th17 differentiation, which synergistically enhances RANKL-dependent osteoclastogenesis. Mφ macrophage, RF rheumatoid factor, CCP Cyclic citrullinated peptide, M-CSF macrophage colony-stimulating factor

Rheumatoid arthritis (RA) is a typical autoimmune disease characterized by chronic inflammation and synovial hyperplasia, eventually leading to bone destruction.^73^ Pathologically, the autoimmunity of RA is associated with the presence of disease-specific anti-citrullinated peptide autoantibodies—rheumatoid factor (RF) and antibodies against cyclic citrullinated peptides (CCP).^74^ Studies have found that there are amounts of IL-17, as well as activated CD4^+^ T cells and DCs in the synovial fluid of RA.^75,76^ Following the citrullination of amino acid residues, matrix heterogeneity in the synovial fluid of RA becomes stronger, and DCs infiltrating to the lesion area activate CD4^+^ T cells through antigen presentation and thereby cause them to release soluble RANKL.^77^ Subsequently, damaged bone structure is resorbed by OCs to form residual bone debris, which can further recruit more iDCs.^37,78,79^ Activated Th17 cells release large amounts of IL-17 and stimulate bone stromal cells to secrete RANKL, which together promote the fusion of IL-17RA receptor-induced iDCs to form DDOC.^80^ The DCs-T cells interaction and the closed loop of DCs-OCs induce severe bone destruction,^37,78,79^ and contribute to the development of RA^16^ (Fig. 3). Thus, researchers consider that DCs play a key role in the initiation and maintenance of RA.^75,76^

Physiologically, there are two main subsets differentiated from CDPs: cDCs and pDCs.^27,28^ However, there is another population of CD14^+^ CD1a^+^ CD1c^+^ DCs in RA synovial fluid (Fig. 3), called infDCs, also known as monocyte-derived dendritic cells (Mo-DCs), which are mainly differentiated from monocytes recruited to the site of inflammation.^36^ InfDCs also exhibit a phenotype that results in a broad and unusual range of C-type lectin receptors being expressed, such as isoforms of CD209 and CD206, CD303 and CD207, as well as the lysosomal protein CD208 and lymphocyte-specific protein 1 (LSP-1).^81,82^

When Mo leave the blood and infiltrate inflammatory synovial tissue, the process of Mo-to-DCs transition is influenced by a variety of environmental factors. Among these environmental factors, four appear to be particularly relevant in the context of RA^83^: (1) the dietary metabolites that are agonists of the aryl hydrocarbon receptor (AhR); (2) the extracellular acidosis; (3) the GM-CSF produced by synovial CD4^+^ T cells; and (4) the synoviocytes and the synovial fluid. A study by Reynolds et al. found that GM-CSF-producing T cells were significantly increased in RA synovial fluid, compared with synovial fluid from non-RA inflammatory arthritis, active RA peripheral blood and healthy donor peripheral blood.^84^ Furthermore, after co-cultured with CD4^+^ T cells in RA synovial fluid, but not healthy donor peripheral blood, a subpopulation of Mo differentiated into GM-CSF-dependent CD1c^+^ infDCs. More importantly, these infDCs exhibited potent alloproliferative capacity and enhanced GM-CSF, IL-17 and IFN-γ produced by CD4^+^ T cells. These results demonstrate a therapeutically tractable feedback loop whereby GM-CSF secreted by RA synovial CD4^+^ T cells promotes the differentiation of infDCs with potent capacity to induce GM-CSF-producing CD4^+^ T cells.^84^

In terms of function, infDCs play a critical role in the pathogenesis of RA, since they have the ability to stimulate the production of Th17 cells.^85^ When co-cultured with CD4^+^ T cells, these infDCs can specifically induce T cells to produce IL-17 through the secretion of TGF-β, IL-1β, IL-6, and IL-23.^36^ Besides, infDCs secrete amounts of pro-inflammatory chemokines (e.g. CXCL8 and CCL3), along with IL-6 and IL-23, which can shift the Treg-Th17 balance towards Th17, subsequently recruiting effector T cells by expressing multiple chemokines (e.g. CCL17, CXCL9, and CXCL10).^86^ The activation of Th17 by DCs may contribute to the pathogenesis of RA through three main mechanisms: (1) IL-17 maintain and amplify local inflammation by acting synergistically with pro-inflammatory factors,^87,88^ e.g, TNF-α and IL-17 had synergistic effects in promoting production of IL-6, IL-8, and granulocyte colony-stimulating factor (G-CSF) in synoviocytes, and versus single inhibition, bispecific anti-TNF-α/IL-17 antibodies showed superior efficacy in inhibiting the development of inflammation and bone/cartilage destruction in arthritic mice^87^; (2) IL-17 induces chondrocyte production of enzymes to degrade cartilage and inhibits chondrocyte-dependent matrix synthesis through production of nitric oxide,^89,90^ thereby dysregulating the RANK-RANKL signaling pathway leading to bone destruction^91^; (3)Th17 cells downregulate plasma cell-expressed sialic acid transferase ST6GAL119 through secretion of IL-21 and IL-22,^92^ resulting in a shift from normally glycosylated non-pathogenic anti-citrullinated protein antibodies (ACPA) to insufficiently glycosylated ACPA with pro-osteoclastogenic activity.^93,94^

Not only that, under inflammatory pathological conditions, infDCs can directly convert into OCs to induce severe bone destruction,^95^ which is promoted by protein citrullination and ACPA binding to DCs.^77^ The description of the DCs/OCs transdifferentiation is detailed below—part of *Osteoclast.* Similar to infDCs, cDCs contribute to the development and persistence of RA. CD1c^+^ cDCs present in synovial fluid from patients with RA are characterized by increased expression of proinflammatory cytokines and a heightened ability to induce pathogenic IFN-γ^+^ IL17^+^ CD4^+^ T cells in vitro.^96^ However, pDCs appear to play a protective role in RA. Although a large subpopulation of pDCs has been found in the RA synovial tissue,^97^ depletion of pDCs instead exacerbated arthritis and inflammatory responses in mice.^98^ Taken together, these findings suggest that the subsets heterogeneity of DCs is enhanced in RA, and the emergence of new subsets during this disease would be better to suit the needs of disease progression.

Apart from subsets heterogeneity, RA alters the distribution of DCs and the functional markers expression. Research has shown a decrease in the proportion of cDCs and pDCs in the blood of RA patients, while the proportion of these cells in the synovial fluid was increased.^99^ Several mechanisms contribute to the reduction of DCs in the bloods. These mechanisms include increased recruitment of DCs to sites of inflammation or lymph nodes, decreased production or release from the bone marrow,^85^ and impairments in the ability of DCs to migrate to draining lymph nodes.^100,101^ For instance, DCs within the RA synovium express elevated levels of CCR6, a receptor for CCL20, a chemokine active on both iDCs and Th17 cells. Notably, CCL20 is located in proximity to iDCs within the synovium, suggesting its critical role in facilitating DCs recruitment into synovial tissue.^100^ On the other hand, DCs in the RA synovium demonstrate downregulation of CCL19 and CCL21, as well as their receptor CCR7, which is associated with impaired migration to lymph nodes.^100,101^

Moreover, the synovial microenvironment in RA induces metabolic and transcriptional reprogramming of DCs.^102^ In contrast to blood CD1c^+^ DCs, synovial CD1c^+^ DCs show a higher expression of many glycolytic genes: e.g., GALM, FABP 5, FBP 1, TGM 2 and ALDOC; as well as metabolic transporter proteins such as SLC25 A19 and the TCA cycle gene SUCNR 1.^102^ The expression of FAAH (involved in fatty acid amide catabolism) and SLC16A4 (facilitating lactate and pyruvate transport), however, was significantly reduced.^102^ In addition, the transcriptional profiling of DCs in RA is altered and has a correlation with disease activity levels.^103^ Single-cell gene expression assays have shown that distinct DCs clusters in RA are characterized by upregulation of TAP1, IRF7, and IFNAR1, indicating that DCs with this cluster profile have an increased capacity to activate T cells, produce type I interferons, and process antigens.^103^ These findings suggest that metabolic or transcriptional profiling of DCs could improve our ability to detect and understand the heterogeneity of RA and guide personalised therapies for autoimmune diseases.

In summary, the distribution, the subsets heterogeneity and alterations in key functions of DCs, such as cytokine secretion and migratory capacity, have been strongly correlated with the onset and progression of RA, leading to tolerance defects and increased inflammation.^85^ As dysregulated DCs have been shown to be involved in RA, there is now a gradual increase in the emerging therapies that exploit or target DCs in two main ways: (1) systemic immunomodulation by selectively targeting the dysregulated functions of DCs (antigen uptake, phenotypic maturation, migration or cytokine secretion) or complete depletion of DCs subsets with key pathogenic roles^104,105^; and (2) antigen-specific therapeutic interventions based on autologous DCs loaded with their own antigens to restore tolerance.^106,107^ The latter mainly involve building the DCs-targeting nanoplatforms and utilizing tolDCs-derived exosomes.^108,109^ However, further investigation is required to determine the mechanism of action and clinical efficacy of these novel strategies.

### Biomaterials-mediated bone repair and regeneration

Osteoimmunomodulation has emerged as a critical concept in the development and evaluation of bone biomaterials.^110^ The creation of immunoregulatory biomaterials to drive immune responses towards tolerance offers the potential to manage early inflammation associated with implanted biomaterials, thereby enhancing tissue regeneration.^111^ Nevertheless, implanted biomaterials themselves could induce DCs maturation through the concurrent production of danger signals and immunoreactive foreign agents.^12^ Thus, understanding the role of DCs in the early immune reactions towards implanted biomaterials could possibly provide novel therapeutic targets for biomaterial-mediated bone repair and regeneration.

Researchers in regenerative medicine acknowledged that excessive and prolonged adaptive immune response is detrimental to tissue regeneration, leading to failed regeneration or foreign body rejection.^110^ Induction of tolDCs, however, can constitute immunoregulatory microenvironment favoring injury healing and tissue repair.^112^ Adu-Berchie and colleagues preliminarily adopted biomaterials as adjuvants carrying immunoregulatory molecules (i.e., IL-10, TGF-β, and corticosteroids) to improve tissue regeneration via inducing Treg-mediated immune tolerance.^17^ However, large-scale immunosuppression can increase the risk of de novo malignancies and infections.^113^ Recent studies have uncovered that biomaterials could influence DCs polarization through their inner properties without carrying immunoregulatory molecules.^17,18^ Specifically, when DCs are exposed to the biomaterials, their phenotypes and functions altered dependent on the physicochemical properties of the biomaterials, such as chemical composition, hydrophobicity, topography, surface protein, spatial structure, and surface potential.^114,115^ The underlying mechanisms by which DCs respond to biomaterials primarily involve integrins, toll-like receptors, C-type lectin receptors, inflammasomes, and the mTOR-NF-κB pathway in autophagy.^115,116^ However, there is limited literature exploring the mechanisms and influencing factors related to DCs-targeted biomaterials aimed at promoting bone repair and regeneration.

Titanium (Ti) represented one of the most well-established materials in dental implantation and orthopedics. Nevertheless, Ti ions, nano/micro-particles, and bacterial antigens cumulated at the implant-bone interface can initiate peri-implant immune response and hamper osseointegration, possibly leading to aseptic loosening, infections, and subsequent bone loss.^117,118^ Human Mo-derived DCs, displaying lower expression levels of surface MHC II, co-stimulatory molecules (CD40, CD80, and CD86), and chemokine receptors (CCR6, CCR7), yet increased response capacity towards allogenic lymphocytes when exposed to Ti^2+^, have an ability of taking in Ti^2+^.^119^

**Fig. 4:**
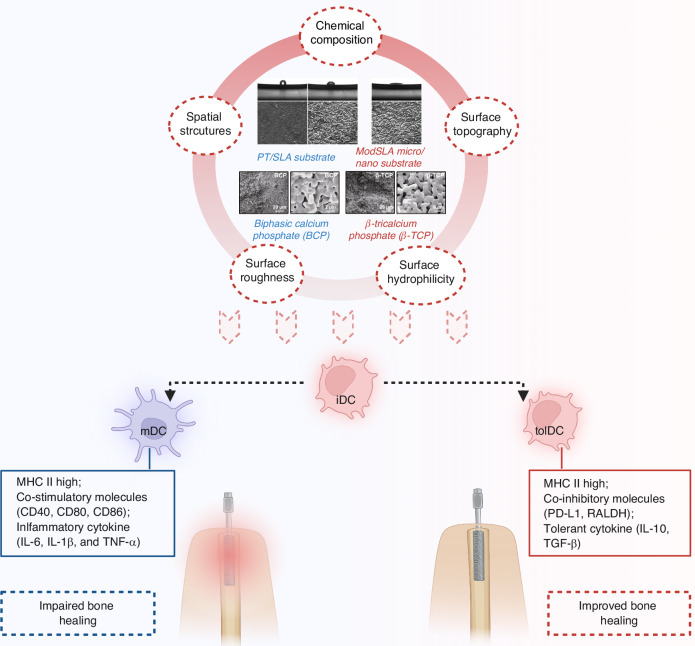
The effects of dendritic cells (DCs) in biomaterials-mediated bone repair and regeneration. The intrinsic physicochemical properties of biomaterials could modulate DCs polarization to influence bone healing and regeneration, such as surface chemistry, hydrophilicity, topography and spatial structures, roughness, and so on. Materials like pure titanium (PT) and sand-blasted, acid-etched titanium (SLA) surfaces promote DCs maturation, characterized by the upregulation of co-stimulatory molecules (e.g., CD40, CD80, CD86) and pro-inflammatory cytokines (e.g., IL-6, TNF-α), which drive immune activation and inflammation, potentially impairing bone healing. In contrast, modified surfaces such as modSLA or β-TCP induce a tolerogenic phenotype in DCs, marked by the downregulation of co-stimulatory molecules and the upregulation of anti-inflammatory cytokines (e.g., IL-10, TGF-β). These tolDCs foster a regulatory immune environment, enhancing osteoblast differentiation and supporting bone regeneration. mDCs mature dendritic cells, iDCs immature dendritic cells, tolDCs tolerogenic dendritic cells

This suggested that Ti ions could alter the phenotype and function of DCs, contributing to an Ti-related immune response and potentially initiating metal hypersensitivity and peri-implantitis.^119^ Additionally, DCs response on different Ti surfaces influences osteogenic capacities. DCs cultivated on the surfaces of pure titanium (PT) and sand-blasted and acid-etched (SLA) developed mDCs phenotypes, while the surface of modified SLA (modSLA) induced iDCs phenotypes and demonstrated superior osteogenic capacities by promoting immune-regulatory microenvironment.^120^ Consistently, Yang et al. reported that when compared to DCs seeded on culture plates, an increase of pro-inflammatory cytokines (i.e., IL-6, IL-12, TNF-α) was observed amidst decrease of anti-inflammatory cytokines (i.e., IL-1ra, IL-4, IL-10) in DCs seeded on PT and SLA surfaces.^121^ DCs cultivated on PT and SLA surfaces displayed inhibited osteogenic differentiation of MC3T3-E1 cells indicated by reduced expression of osteogenic genes (RUNX2, COL1, ALP, and OCN) and decreased ALP activity.^121^ Their findings were in accordance with previous studies by Zheng and colleagues.^122^ DCs treated on modSLA surface, on the contrary, exhibited a potential of promoting osteogenic differentiation of MC3T3-E1 cells with an increase in RUNX2 and ALP expression as well as ALP activity.^122^ Recent studies confirmed that in comparison with microscale SLA, hierarchical micro/nanostructures incline to maintain iDCs phenotypes with lower levels of inflammatory responses by downregulating β2 integrin-FAK signaling, probably favoring the osseointegration process.^21^ Collectively, titanium substrates with different surface modifications could induce altered response of DCs (Fig. 4). These studies seemed to indicate that bone biomaterials with desirable osteogenic capacities incline to induce the immature phenotype of DCs. Nevertheless, current researches concerning the interactions of DCs and OBs are limited to in vitro studies, whether DCs behaviors influence biomaterial-mediated osseointegration remained to be further explored.

DCs also serve as the sentinel for bone grafts-induced immune responses. DCs seeded on porous β-tricalcium phosphate (β-TCP) showed a decrease in co-stimulatory molecules (i.e., CD40, CD86) and pro-inflammatory gene IFN-γ while an increase in anti-inflammatory genes (i.e., IL-1ra, IL-4 and IL-10, and TGF-β1).^22^ In addition, conditioned medium harvested from DCs cultured on β-TCP markedly promoted OB differentiation by increasing the expression of osteogenic genes RUNX2, ALP, as well as OCN.^22^ Meanwhile, osteoinductive biphasic calcium phosphate (BCP) scaffolds lowered T cell response and promoted osteogenesis by inducing M2 macrophage polarization whilst inhibiting DCs maturation.^110^

Despite the progressively recognized role of DCs in immunoregulatory biomaterials, researches concerning their impact on biomaterial-mediated osseointegration were scarce. More importantly, the maturation of DCs seem not to be necessarily for the development of destructive inflammations, and it might be unilateral to determine the prognosis of implanted biomaterials solely based on DCs-biomaterial interactions.^12^ Host reactions towards implanted biomaterials are intricate, with underlying intercellular or intersubgroup alternative/balancing mechanisms. In the future, with in-depth researches and the application of high-throughput approaches, realization on DCs will go further. What’s more, the crosstalk and mechanisms of regulation between bone cells and DCs must be further investigated before DCs become a focus of new clinical therapies involving bone tissue regeneration and repair.

### Others

In addition to the above-mentioned bone-related diseases, DCs are implicated in the development of nonunions, Langerhans cell hyperplasia (LCH), multiple myeloma (MM) and so on. LCH often involves severe bone destruction with DCs monoclonal hyperplasia as a pathological change. Research analyzing LCH bone biopsies has revealed a strong association between LCs and differentiated OCs-like cells, suggesting that abnormal antigen presentation may initiate the pathological activation and differentiation of DCs.^123^ However, the specific mechanism requires further investigation. MM, a myelodysplastic malignant tumor, can release the chemokine SDF-1 to attract iDCs via the corresponding receptor CXCR4 on the their surface.^124^ Additionally, the secretion of soluble RANKL by MM cells promotes the activation and transdifferentiation of DCs into OCs with bone resorption function, leading to erosion of tumor cells in later disease stages.^125^ Nonunion refers to fractures that do not heal properly. ScRNA-seq of intramedullary canal tissue from patients with femoral nonunions showed higher proportions of Mo and CD14^+^ DCs, and lower proportions of T cells, myelocytes, and promyelocytes, compared to native bone samples.^126^ CD14^+^ DCs have been shown to stimulate OCs formation in inflammatory environment,^95^ explaining the failure of bone healing in patients with nonunion due to increased bone resorption.

## Interactions of DCs with other cells in bone microenvironment

### Osteoclast (OCs)

**Fig. 5:**
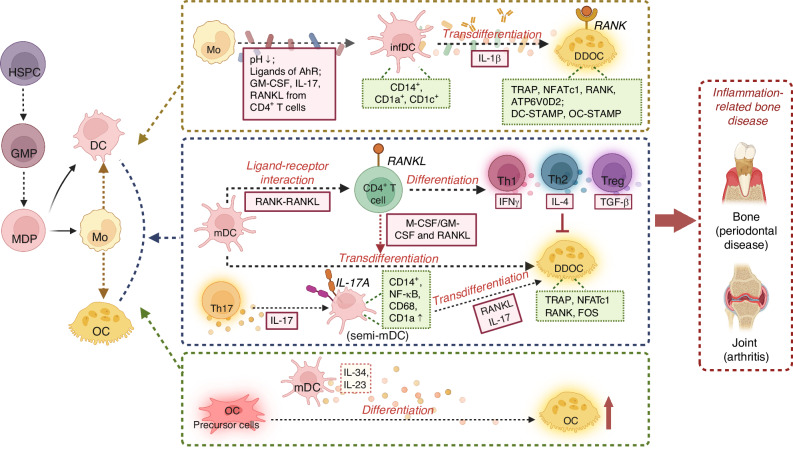
Cellular interactions between dendritic cells (DCs) and osteoclasts (OCs) in pathological conditions. Under inflammatory conditions, such as rheumatoid arthritis and periodontitis, DCs can directly transdifferentiate into OCs, contributing to inflammation-induced bone erosion. This process of DCs-OCs transdifferentiation is modulated through three major mechanisms: (1) In the presence of macrophage colony-stimulating factor (M-CSF) and receptor activator of nuclear factor kappa-Β ligand (RANKL), DCs differentiate into OCs efficiently, as shown in the yellow dashed box. This interaction is a key driver of osteoclastogenesis under inflammatory conditions; (2) RANKL, produced by Th1, Th2, and Treg cells, or IL-17, produced by Th17 cells, can enhance DC-mediated osteoclastogenesis in an inflammatory microenvironment, as illustrated in the blue dashed box; (3) DCs can promote osteoclastogenesis by secreting osteoclastogenic cytokines, such as IL-23 and IL-34, which facilitate OC differentiation, as shown in the green dashed box. HSPCs hematopoietic stem and progenitor cells, GMP granulocyte-macrophage progenitor, MDP myeloid progenitor cells, Mo monocyte, DDOC dendritic cell-derived osteoclast

OCs played distinguishable roles in manipulating bone remodeling, with extensive heterogeneity and plasticity.^37^ Inflammation-related bone destruction was observed when the metabolism of OCs were disequilibrated, as shown for example of RA,^37^ periodontitis^127^ or postmenopausal osteoporosis.^128^ Recently, some studies have revealed that DCs can directly transdifferentiate into OCs under pathological conditions, leading to inflammation-induced bone erosion; and residual bone debris formed by OCs can further recruit more DCs.^37,78,79^ DCs-OCs interaction was predominantly modulated in three manners (Fig. 5): (1) In the presence of macrophage colony-stimulating factor (M-CSF) and RANKL, DCs can efficiently differentiate into OCs, known as DDOC^95^; (2) RANKL^+^ T cell and IL-17-producing Th17 cells can facilitate DCs-mediated osteoclastogenesis in inflammatory conditions^80,125^; (3) DCs can promote OCs differentiation via secretion of osteoclastogenic cytokines (IL-23, IL-34).^129,130^ Together, coupling reactions between DCs and OCs further unveiled their interactive and reciprocal roles in manipulating bone homeostasis, providing potential therapeutic targets for OCs-mediated bone destruction diseases.

There are several lines of evidence supporting the possible link between DCs and OCs. Firstly, DCs and OCs both developed from hematopoietic stem cells (HSCs), of which Mo were the common precursors.^131^ Expression levels of the most relevant genes involved in OCs function (i.e., TRAP, β3-integrin, NFATc1, RANK, and ATP6V0D2) in Mo-derived OCs and DDOC showed highly similarity.^37^ Interestingly, osteoclastogenesis seemed to reprogram fewer genes when derived from DCs (4 073 genes) than from Mo (7 798 genes), and the cluster of OCs and intermediate stages were twice as close to DCs than to Mo.^132^ Secondly, same surface markers such as dendritic cell-specific transmembrane protein (DC-STAMP) have been identified in DCs and OCs,^133^ and expression pattern of which is essential with regards to fusogenic and osteoclastogenic potential.^134^ DC-STAMP plays an imperative role in bone homeostasis by modulating the differentiation of OCs and OBs,^135^ which also provides theoretical cornerstones for DCs-OCs transdifferentiation.^12^

DCs-OCs transdifferentiation from Mo/Mφ precursor cells showed high plasticity depending on local factors and stimuli in the milieu. While DCs were traditionally regarded as terminally differentiated cells, recent studies have indicated that their differentiation can be further modulated by the surrounding microenvironment.^136^ In the presence of M-CSF and RANKL, human peripheral blood Mo-derived DCs can transdifferentiate into OCs in vitro in a manner more efficient than Mo-OCs transdifferentiation with regard to fusion rate and nuclei size.^95^ DCs express the M-CSF receptor colony-stimulating factor 1 (CSF-1), and 50%–70% of CD11c^high^ DCs are absent in the osteopetrotic CSF-1^-/-^ (op/op) mice, indicating that DCs subsets are derived from M-CSF-dependent common progenitor pool including OCs precursors at least under inflammatory conditions.^11^ Furthermore, the DCs-OCs transdifferentiation involved pro-inflammatory cytokines such as IL-1β or TNF-α, as well as components of the extracellular matrix (ECM) such as hyaluronic acid, which can be greatly enhanced by adding the RA synovial fluid.^95^ Recent evidence suggests that DCs-OCs transdifferentiation is correlated with protein citrullination, a post-translational modification catalyzed by peptidyl arginine deiminase (PAD) enzymes, as well as with RA-specific ACPA.^77,92^ An in vitro study observed that increased PAD activity and elevated levels of protein citrullination in DCs potentially facilitated their development into OCs.^77^ ACPA were found to enhance OCs differentiation from Mo-derived or circulating CD1c^+^ DCs by promoting the release of IL-8.^77^ Importantly, blocking IL-8 binding or inhibiting PAD enzymes completely abolished the stimulatory effects of ACPA and further diminished OCs development, indicating a crucial role for protein citrullination and ACPA in the DCs-OCs transdifferentiation.^77^ However, there is currently no clear evidence supporting the physiological relevance of this phenomenon in vivo. Together, these finding offer a potential strategy to limit OCs development and promote bone regeneration in ACPA-positive RA individuals via modulating DCs.

In addition, DCs could contribute to inflammation-induced osteoclastogenesis during immune interactions with CD4^+^ T cells through RANK-RANKL signaling.^39^ It’s noteworthy that beside of OCs, RANK is also expressed on DCs, and its binding with CD4^+^ T cell-derived RANKL can lead to NFATc1-mediated osteoclast-related gene transcription in the DCs, contributing to DDOC formation.^125^ Alnaeeli and colleagues uncovered that immune interactions with CD4^+^ T cells rendered murine BM-derived CD11c^+^ DCs develop into TRAP^+^ CT-R^+^ Cathepsin-k^+^ OCs in a RANKL/RANK-dependent manner.^137^ In addition to RANKL^+^ T cells, Th17 cells and their specific cytokines IL-17 also facilitated DCs-mediated octeoclastogenesis. IL-17A produced by activated Th17 cells induced a semi-mature phenotype of CD14^+^ DCs with expressing NF-κB, CD68, CD1a, MHC II and CCR6 and induced cell fusion.^80^ IL17A-transdifferentiated iDCs in MM patients showed increased transcription of OCs-maturation genes (i.e., NFATC1 and FOS), which was explainable for the progressive bone destruction.^138^ Taken together, these studies emphasized the involvement of RANKL^+^ T cells under inflammatory conditions and IL-17-producing Th17 cells in DCs-derived osteoclastogenesis.

Furthermore, mDCs can promote osteoclastogenesis via secretion of related cytokins. IL-23 is a heterodimeric cytokine produced predominantly by macrophages and DCs, which has been demonstrated to upregulate RANK expression of myeloid precursor cells in a dose-dependent manner and thereby indirectly promotes osteoclastic differentiation.^129^ Additionally, DCs can also produce osteoclastogenic cytokine IL-34, systemic administration of which intensively induced OCs differentiation and reduced bone mass in mice.^139^

Above all, hematopoietic stem/progenitor cells represent key origins of OCs under homeostasis, whereas OCs can further differentiate from DCs in inflammatory-related bone diseases. The plasticity of OCs development suggested its flexibility and adaptation under different pathological conditions.^37,125^ Based on the studies, DDOC can serve as a novel subgroup of OCs, manipulated by the interactions of DCs, T cells, bone myeloid cells, and bone forming cells under different inflammations.^39^ Delineating the mechanisms underlying DCs-OCs transdifferentiation will support the new paradigm of osteoimmunology, and facilitate novel therapies in osteoclastogenesis-related bone erosion diseases.

### T cells

Among bone marrow cells, there are about 3% ~ 5% T cells.^140,141^ Bone marrow is a major reservoir for central memory T cells,^142^ and BMDCs are thought to be advantageous for triggering central memory T cells-mediated responses with antigen-dependent contacts.^143^ Recent research reveals that T cells play a particularly key role in bone remodeling and bone regenerative,^126^ and depletion of T cells results in stiffer bones with greater fracture propensity^144^ or nonunion.^126^ Moreover, T cells also play a critical role in inflammation-induced osteoclastogenesis and subsequent bone loss.^11,145^

**Fig. 6:**
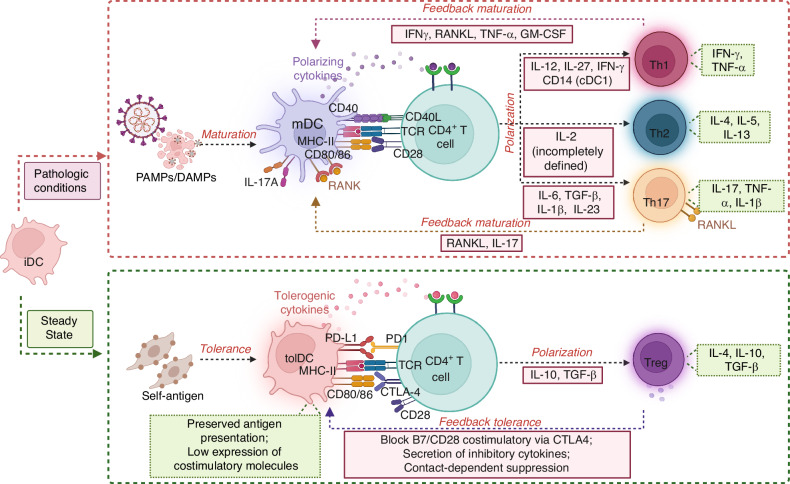
Cellular interactions between dendritic cells (DCs) and T cells in bone remodeling and immune responses. DCs activate naive CD4^+^ T cells and promote effector differentiation in response to immune perturbations. This process involves a coordinated series of signals: (1) processed antigen presented on major histocompatibility complex (MHC) molecules to antigen-specific T cell receptors (TCRs), (2) recognition of costimulatory signals on DCs (e.g., CD80, CD83, CD86) by receptors on T cell surfaces (e.g., CD28, CD40, CTLA-4), and (3) instructive cytokine and cell surface signals (e.g., IL-12, IFN-γ) that guide differentiation. Th1 cells promote bone resorption by secreting osteoclastogenic factors, such as RANKL and TNF-α. In contrast, Th2 cells generally support bone homeostasis by inhibiting osteoclastogenesis. Th17 cells, known for their role in inflammation-induced bone loss, can mature DCs via the RANK/RANKL signaling pathway, but they can also suppress DCs maturation through IL-17 secretion, leading to bone resorption. When exposed to harmless antigens, immature DCs (iDCs) differentiate into tolerogenic DCs (tolDCs) with preserved ability of antigen presentation but featured low expression of costimulatory molecules, leading to regulatory T cells (Treg) polarization and bone homeostasis. DAMP damage-associated molecular pattern, PAMP pathogen-associated molecular pattern, TGF-β transforming growth factor-beta

Th1, Th2, Th17 and Treg are main subsets differentiate from naïve CD4^+^ T cells in chronic inflammatory bone disease such as RA and periodontitis.^146^ The differentiation of each Th subsets requires a series of signals during antigen presentation, and the complexity of the Th cell response depends on the immune states of DCs (Fig. 6). A coordinated series of signals is needed for successful antigen presentation, including (1) processed antigen on major histocompatibility complex (MHC) molecules (HLA in humans) to antigen-specific TCRs, (2) recognition of costimulatory signals exhibited on DCs (e.g., CD80, CD83, CD86) by their receptors on T cells surface (e.g., CD28, CD40), and (3) instructive cell surface and cytokine signals (e.g. IL-12, IFN-γ).^115^

In addition to the classical molecules mentioned above, DCs can also regulate the differentiation of T cells through unique cytokines in the bone environment. Th1 polarizing cytokines involve IL-12, IL-27 and IFN-γ, among which IL-12 is supposed to be the primary Th1 differentiation cytokine and is mainly produced by cDC1.^147,148^ Besides, strong TCR signaling^149,150^ and strong co-stimulatory signaling^151^ also favor the generation of Th1 cells. Under infection or inflammation conditions, Th1-derived IFN-γ feedback on DCs to further promote IL-12 production^152^ and promotes T cell secretion of osteoclastogenic factors RANKL and TNF-α, thus promotes OCs formation and bone resorption.^153^

Accumulating evidence supports that cDC2 are major DCs subset implicated in Th2 differentiation in skin,^154^ lungs,^155,156^ and intestinal tract.^157^ Recently, cDC2 were also found accumulating in a mature state in the RA synovium, where they induce more potent Th2 type responses.^158,159^ Th2 cells produced cytokines, including IL-4, IL-5 and IL-13,^160^ which have been reported to have anti-osteoclastogenic functions in vivo.^15,161^ To elucidate the potential mechanisms by which DCs drive Th2 cells development, several models have been proposed.^162^ One prevailing model indicates that the expression of the transcription factors interferon regulatory factor 4 (IRF4) and Krüppel-like factor 4 (KLF4) by DCs is necessary for the induction of Th2 cells.^163–165^ Another model proposes that the ‘third signal’ driving Th2 cell responses may be the absence of polarizing cytokines, implying that adequate TCR signals and CD28 co-stimulation can proceed Th2 cells differentiation in the absence of any other extra stimulus or cytokines.^166,167^ However, no consensus has been reached to date.

Th17 cells have been highlighted in the pathogenesis of bone loss disease since their discovery,^15,146,168,169^ due to that IL-17 triggers osteoclastogenesis^170^ and synergizes potently with TNF-α and IL-1β.^171^ The differentiation of Th17 cells from naïve CD4^+^ T cells is initiated by stimulation with professional antigen-presenting cells and polarizing cytokines involving IL-6, TGF-β, IL-1β, and IL-23.^169,172^ Strong TCR stimulation in concert with certain microbial stimuli could increase IL-6 production from DCs and Th17 polarization.^169^ Except for the classic IL-6 signaling pathway, IL-6 trans-presentation in IL-6Rα-expressing DCs could also induce the pathogenic Th17 cell differentiation through gp130 molecules expressed on T cells.^173^ What’s more, in the synovial fluid from RA patients, IL-23 is the prominent polarizing cytokine involved in the Th17 cell responses induced by infDCs.^36^

RANK/RANKL system is another important regulators of interactions between DCs and T cells in the bone environment, independent of CD80/86.^174^ RANK expression appears to be restricted to mDCs,^75^ while activated T cells, Th17 cells in particular, are essential source of RANKL.^175^ DCs localized in the bone stroma can aggregate with T cells to form inflammatory foci, where migrate through RANK-RANKL system.^12^ The expression of RANK on the surface of DCs during inflammation of bone tissues indirectly induces bone degradation through regulating activity of T cells and survival of OCs.^176,177^ The RANK/RANKL system can not only provide co-stimulation required for CD4^+^ T cell priming in the absence of CD40/CD40L,^178^ but also provide further activating and survival signals to DCs.^179,180^ RANKL secreted by CD4^+^ T cells also promotes DCs upregulate the expression of pro-inflammatory cytokines such as IL-6, IL-1β and T cell differentiation factors such as IL-12 and IL-15.^181^ Besides, immune interactions with CD4^+^ T cells contributes to the development of DCs into OCs in a RANKL/RANK-dependent manner, inducing bone resorption after mice calvarias adoptive transfer.^137^ Notably, RANKL and CD40L have functional similarity but predominate at different stage. CD40L-CD40 signaling is more prominent during the initiation and effector phases, while RANKL-RANK signaling act at later times to guarantee the T memory formation and wind down remaining T cells-DCs interactions through upregulation of OPG.^7,182^

When meet harmless antigens such as self-antigens, DCs differentiate into tolDCs with preserved ability of antigen presentation but featured low expression of costimulatory molecules and pro-inflammatory cytokines such as IL-1β, IL-6, TNF-α,^183,184^ leading to T cell responses inhibition and Treg production.^185,186^ In experimental RA, tolDCs was observed to not only abrogate activation and proliferation of both naïve and pre-activated CD4^+^ T cells, but also potentially active Tregs.^187^ Treg shows a beneficial impact on bone remodeling by secreting anti-inflammatory cytokines IL-4, IL-10, and TGF-β that inhibit ostroclastogenesis and promote bone formation.^175^ Treg can also act on DCs in return and dampen the stimulatory properties of DCs through multiple mechanisms,^148^ among which cytotoxic T-lymphocyte-associated protein 4 (CTLA-4) expressed on T cells has received considerable attention. CTLA4-Ig can not only act as a co-stimulation modulator to block CD28 costimulatory,^188–190^ but also modify DCs from collagen-induced RA mice into tolDCs to increase the Treg population through in a TGF-β-dependent manner.^191^ Moreover, Tregs potently suppress the autophagy of DCs in a CTLA4-dependent manner relating to the activation of the PI3K/Akt/mTOR axis and FoxO1 nuclear exclusion, and CTLA4-Ig renders DCs less immunogenic in RA via diminishing autophagy.^192^ In addition, contact-dependent suppression is another mechanism independent of MHC-II molecules,^193^ referring to the tight adhesion between Treg cells and DCs which forms an occupancy-based inhibitory network to reduce the ability of DCs to engage other T cells.^194^ What’s more, the secretion of inhibitory cytokines forms a immunosuppressive milieu, promoting the mutual interplay between Tregs and tolDCs.^195^ Thus, the bidirectional interaction between tolDCs and Treg cells constitute the “tolerogenic feedback loops”.^185^

### B cell

The bone marrow microenvironment formed a nurturing network for B lymphopoiesis and developing,^196^ while B cells influenced bone homeostasis in terms of OB and OCs differentiation in turn,^197,198^ generating a codependency relationship. Recent studies unveiled that B cells manipulated a series of inflammatory bone diseases, including fracture healing,^199^ periodontitis,^200^ postmenopausal osteoporosis,^201^ RA^202^ and MM,^203^ while DCs play a significant role in the adaptive immunity initiated by B cells.^204,205^

**Fig. 7:**
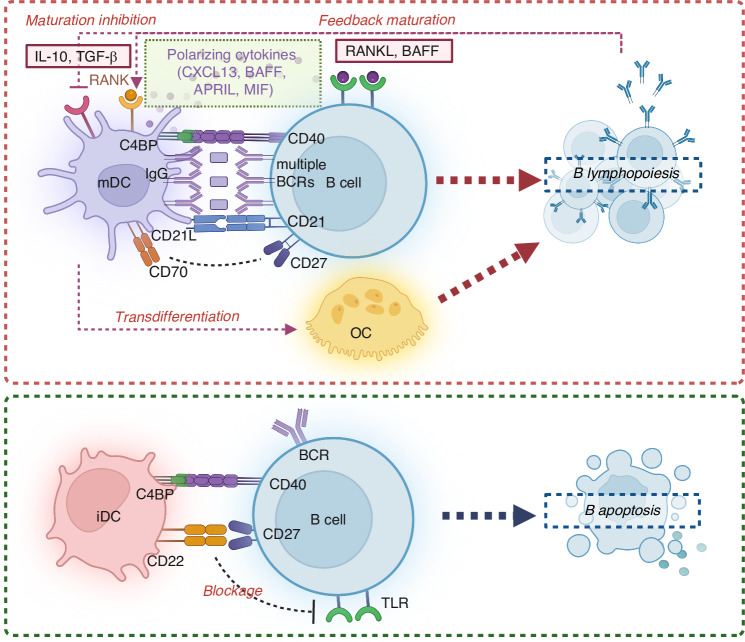
Cellular interactions between dendritic cells (DCs) and B cells. DCs play a crucial role in the adaptive immune responses initiated by B cells, with B cells receiving immune signals from DCs via multiple B cell receptors (BCRs) on their surface, in contrast to the way DCs interact with T cells. The antigen presentation by DCs to B cells involves: (1) a variety of antigen forms held on DCs, including immune complexes of antigen, antibodies, and complement; (2) unique co-stimulatory signaling molecules, such as B cell activation factor (BAFF), A proliferation-inducing ligand (APRIL), and macrophage migration inhibitory factor (MIF); (3) immunoregulatory cytokines, including C-X-C motif ligand 13 (CXCL13), IL-6, TNF-α, and other cytokines that modulate inflammatory or tolerogenic responses. Additionally, direct cell-to-cell interactions such as CD40-CD40L signaling, and soluble factors secreted by DCs also influence B cell activation, survival, and differentiation in the bone marrow microenvironment. Given the limited interaction between DCs and B cells within the bone marrow environment, the mechanisms outlined above were primarily derived from studies examining the interactions between follicular dendritic cells (fDCs) and B cells. IgG immunoglobulin G

Unlike the way DCs interacting with T cells, B cells receive immune signals from DCs via the multiple BCRs on their surface^206^ (Fig. 7). Beyond that, the features of antigen presentation by DCs to B cells lie in (1) diversified forms of antigen held on DCs, involving immune complexes of antigen (ICs), antibody and complement^207,208^; (2) Unique co-stimulatory signaling molecules, such as C4b-Binding Protein (C4BP) and CD21L^206,207^; (3) Immunoregulatory cytokines, including B lymphocyte chemoattractant (BLC/CXCL13), B cell activation factor of the TNF family (BAFF), and macrophage migration–inhibitory factor (MIF).^33,206,209,210^ The mechanisms outlined above were primarily derived from studies examining the interactions between follicular dendritic cells (fDCs) and B cells. fDCs represent a unique population of cells that is essential for efficient germinal centre (GC) formation,^211^ and they are not derived from HSCs but rather from follicular basal cells.^212^ Functionally, both fDCs and traditional DCs (derived from HSCs) serve as antigen-presenting cells; however, fDCs do not express MHC II and are characterized by a cell surface abundant in antibody receptors, allowing them to directly adsorb natural antigens, typically those captured by complement or antibodies.^212^ In contrast, traditional DCs must phagocytose antigens and process them into peptide fragments for presentation on MHC molecules. Given the limited interaction between DCs and B cells within the bone marrow environment, future research could benefit from exploring existing findings on the interactions between fDCs and B cells to achieve a more comprehensive understanding, as several possible mechanisms have been proposed.

DCs can directly influence B cell response through the production of soluble factors. DCs are a major source of a TNF family cytokine BAFF in the early phase of RA disease, which leads to the enhanced activation, proliferation, and differentiation of B cells.^213^ BAFF-signaling pathway blocked by a novel BAFF antagonist resulted in not only suppressed B cell activation, but also inhibited DCs generation associated with regulatory B cells, providing potential treatment of RA.^214^ A proliferation-inducing ligand (APRIL) is another member of TNF family secreted by DCs,^215^ acting as a key modulator of B cell development, differentiation, and survival.^216^ APRIL shares a similar structure with BAFF and also binds to transmembrane activator and calcium modulator and cyclophilin ligand interactor (TACI) and B cell maturation antigen (BCMA) to activate the NF-κB, PI3K/Akt and MAPK pathways, thus promoting B cell proliferation and survival in bone marrow microenvironment.^217^ Besides, DCs also promote the survival of recirculating B cells in the bone marrow via the production of MIF while the conditional ablation of BMDCs led to the specific loss of recirculating mature B cells.^33^

DCs can also affect B cell response via direct cell-to-cell interaction. DCs were implicated in presenting antigens as ICs to B cells with multiple BCRs engaged simultaneously, subsequently inducing T cell-independent B cell activation.^207,208^ In vitro study showed that DCs provided B cells with survival signals in the presence of CD40L on DCs interacting with CD40 on B cells.^218^ Recently, a distinct subset of pDCs characterized by the expression of CD2, CD5, and CD81 on the surface has been found in the bone marrow.^219^ They were found to be the potent stimulators of B cell activation and antibody production, in which the cell-cell contact via CD70 plays a critical role.^219^ The optimal suppression of B cell activation by immature BMDCs was dependent on the BCR co-receptor CD22 in a contact-dependent manner.^220^ Presence of immature BMDCs enhanced the Ag-induced bone marrow B cells apoptosis on BCR-dependent manner, suggesting that DCs in the bone marrow might play a role in B cell negative selection.^221^

DCs can regulate B cells response indirectly through other immune cells. In bone marrow, cDCs was found to rescue B lymphopoiesis indirectly through transdifferentiating into functional OCs which control the bone microenvironment and modulate B cell development.^222^ B lymphopoiesis was blocked at the pro-B to pre-B transition in the bone marrow of ostropetrotic oc/oc mice,^223^ while oc/oc mice received intraperitoneal injection of cDCs rescued OCs function, leading to restoration of B cells developments.^222^

In addition to antibody production and effector function, B cells are also known to be potent regulators of immune responses mediated by DCs. Activated B cells overexpress RANKL in the context of inflammatory bone diseases such as RA^224^ and periodontal disease,^225^ which influence DC response as mentioned in the part of *Periodontitis and Rheumatoid arthritis*. When under microenvironment of malignancy such as MM, myeloma cells secreted immunological inhibitory cytokines such as TGF-β1 and IL-10 which were responsible for deficient CD80/86 upregulation during DCs maturation and were taken accountable for defective DCs differentiation.^226,227^ Nevertheless, studies on interactions between DCs and B cells in bone microenvironment are still lacking, calling for further research to explore the underlying mechanisms and the potential therapeutic target.

### Mesenchymal stromal stem cells (MSCs)

Mesenchymal stromal stem cells (MSCs) have exerted their reparative as well as immunoregulatory effects in innate and adaptive immune system.^228,229^ Current researches preliminarily uncovered that MSCs could interact with DCs to alleviate graft rejection and promote tissue repair and regeneration,^228,230^ which have a great potential in bone tissue engineering therapy.

Bone marrow mesenchymal stromal stem cells (BMSCs) have been profoundly applied in bone tissue engineering, concerning its multilineage differentiation potentials in adipogenesis, osteogenesis, and chondrogenesis.^231^ Recent investigations have uncovered potent immunoregulatory properties possessed by undifferentiated BMSCs, which can further inhibit DCs differentiation, maturation, and function.^232^ And chondrogenically differentiated BMSCs also lacked immunogenicity and did not trigger immunogenic responses from DCs in vitro or further induce DCs-mediated T cell activation.^233^ Kiernan and colleagues reported that allogeneic chondrogenic human BMSC pellets did not affect the expression of CD80, CD86, or HLA-DR on iDCs or LPS-matured DCs, where antigen-uptake or migration capacity of the DCs remained unchanged over time.^233^ Nevertheless, Chen and colleagues reported that instead of undifferentiated BMSCs or osteogenic- and adipogenic-differentiated BMSCs, chondrogenically differentiated BMSCs induced DCs maturation with upregulated expression of CD83, whereas no significant difference can be detected in terms of HLADR, CD14, and CD1a.^234^ Similarly, another study found that MSCs did lose their immunosuppressive properties to some degree after chondrogenic differentiation, as indicated by higher surface expression levels of MHC-I, MHC-II, CD80 and CD86, and proliferation in CD4^+^ T-cell populations.^235^ Thus, immunogenicity of chondrogenically differentiated BMSCs is still controversial. Modulation of osteogenically differentiated BMSCs on DCs is dependent on the routes of administration.^236^ Trabanelli and colleagues reported that when compared with detached OCs, adherent BMSCs implanted on scaffolds strongly suppressed DC maturation via a potent downregulation of CD80 and CD86, as well as further inhibited DC induction of CD4^+^ CD161^+^ CD196^+^ T cells via IL-6/TGF-*β* regulation.^236^ DCs migration to the lymph nodes is dependent on the upregulation of chemokine receptor CCR7 and downregulation of tissue anchoring protein E-cadherin.^237^ By inhibiting the expression of CCR7 and ameliorating E-cadherin down-regulation, BMSCs can prevent DCs migration to the lymph nodes and avoid graft rejection.^237^ Taking together, BMSCs can improve transplantation via modulating DCs-mediated T cell activation, while the complicated interplay between BMSCs and DCs and underlying mechanisms are still under research.^228^

In addition to BMSCs, periosteum-derived mesenchymal stem cells (PCs) can potentially represent an oral tissue-engineering therapy for the ease of collection, ameliorated operation injuries, and rapid in situ engraftment.^238,239^ Dai et al. reported lower DCs numbers, lower expression levels of IL-12p35 and -p40 as well as pro-inflammatory cytokine (IFN-γ and TNF-α) of DCs, and upregulated DCs expression of IL-8 and IL-10 after co-culturing with both undifferentiated and osteogenically induced PCs.^230^ Together, these results indicated an overall immunosuppressive effect of PCs on DCs maturation.

### Hematopoietic stem and progenitor cells (HSPCs)

Recent study has confirmed the critical role of BMDCs in HSPCs trafficking, thereby contributing to the regulation of hematopoiesis and establishment of the stem cell niche.^13^ Specific ablation of cDCs after DT treatment in Zbtb46^dtr^ induced HSPCs mobilization, in a manner significantly more efficient than Cd169dtr bone marrow chimeras where Mφ were ablated.^13^ Ablation of cDCs was dependent on upregulated CXCR2 expression in sinusoidal endothelial cells, and of which neutralizing via CXCR2 ligands (CXCL1 and CXCL2) markedly attenuated HSPCs mobilization.^13^ Collectively, BMDCs regulated HSPCs trafficking, probably through manipulation of sinusoidal CXCR2 signaling and vascular permeability.

In addition, a majority of BMDCs resided perivascularly, with high expression of chemokines and TLRs, suggesting its role in sensing and manipulating the hematopoietic response to microbes and inflammatory signals.^13^ In a recent study, multipotent HSCs can sense the alterations in the immunological microenvironment through TLR1/2-mediated stimulation of BMDCs, which induced the secretion of inflammatory cytokines (i.e., IL-1β) into the perivascular niche, and in turn, the regulation of multipotent HSCs, forming a loop.^240^

## Conclusions and perspectives

The past decade has witnessed remarkable expansion in the connotation of osteoimmunology while bone-derived regulation of the immune system is progressively unveiling.^241^ Being the most potent antigen-presenting cells responsible for the activation of quiescent T cells and the orchestration of the immune response, DCs have emerged as a critical component of the bone-immune interface.^11,12^ This is evidenced by several key aspects: (1) their contribution to the regulation of hematopoiesis and the establishment of the stem cell niche in the bone morrow^13,240^; (2) their transdifferentiation into OCs in the presence of specific cytokines (e.g., RANKL and M-CSF) to exacerbate osteolysis, wherein T cells act as a balance switch through the RANK-RANKL signaling^95,125^; 3) their activation of T/B cells that produce cytokines and soluble factors during the bone remodeling^142,213^; 4) their interaction with skeletal cells (i.e., BMSCs, OBs) to participate in the bone repair and regeneration.^110,228,230^ In summary, bone and immune cells coexist within the same microenvironment, interacting with one another across various physiological and pathological contexts. Among these interactions, DCs emerge as key players in the field of osteoimmunology. A comprehensive understanding of the crosstalk between DCs and other cell types is essential for a deeper insight into bone homeostasis and the pathogenesis of bone-related diseases.

However, the majority of studies predominantly focus on models of periodontitis and RA, with relatively few investigations addressing other bone-related disease models. Moreover, in vitro experiments frequently lack thorough in vivo validation and exploration of the underlying mechanisms. The direct interactions and regulatory mechanisms between skeletal cells and DCs also remain underexplored. Although there is a gradual increase in the application of DCs-based immunotherapies, such as DCs-targeted vaccines and epigenetic reprogramming, further research is necessary to elucidate the mechanisms of action and clinical efficacy of these innovative strategies. Additionally, these technologies currently do not integrate with bone regeneration approaches. Modifying biomaterial surface to endow DCs with immunomodulatory properties is expected to provide novel strategies for future orthopaedic surgery, trauma surgery, and implant repair in bone tissue engineering.^14^ The incorporation of biomaterials targeting DCs into immunomodulatory therapeutics has significant potential in the tissue engineering and regenerative medicine.^32,115^ However, most of these research has yet to be translated into the clinic. Despite the abundance of knowledge we possess concerning Mo/Mφ-biomaterials interactions, there is currently a limited understanding of the dynamics between DCs and biomaterials.^111^ Nevertheless, the union of various high-throughput techniques, such as ScRNA-seq and spatial transcriptomics technologies, provide a plethora of opportunities to explore the connection between DCs and osteoimmunology in greater depth.^242^

## Supplementary information

Supplemental material
