# Supplementary material for: Unraveling the mechanisms of bone diseases: targeting dendritic cells in osteoimmunology for internal homeostasis balance
Source: Bone Res. 2025 Sep 28;13:81. doi: 10.1038/s41413-025-00456-7 (PMC12476454; doi:10.1038/s41413-025-00456-7)
Supplement: Supplementary file 1 — Supplemental material [file 41413_2025_456_MOESM1_ESM.docx]

**Supplementary tables**

**Table S1. Abbreviation**

| Abbreviation | Definition |
| --- | --- |
| RANKL | Receptor activator of nuclear factor-κB ligand |
| GM-CSF | Granulocyte macrophage-colony stimulating factor |
| TGF-β | Transforming growth factor-beta |
| IL-17 | Interleukin-17 |
| TNF-α | Tumor necrosis factor-alpha |
| F4/80 | Adhesion G protein-coupled receptor E1, ADGRE1 |
| Ly6c/g | lymphocyte antigen 6 family member C/G |
| XCR1 | X-C motif chemokine receptor 1 |
| DC-SIGN | CD209 antigen-like protein E |
| IFN-γ | Interferon gamma |
| LPS | Lipopolysaccharides |
| CXCL9 | C-X-C motif chemokine ligand 9 |
| CCL17 | C-C motif chemokine ligand 17 |
| CCR7 | C-C motif chemokine receptor 7 |
| RUNX2 | RUNX family transcription factor 2 |
| COL1 | Collagen type I |
| ALP | alkaline phosphatase |
| OCN | osteocalcin |

**Table S2. Different CD molecules of DCs for identification or other** **functions**

| CD molecules | Type of CD molecules | Functions |
| --- | --- | --- |
| CD11c | Surface markers of DCs | Identification |
| CD103, CD11b, CD115 | Special surface markers of BMDCs | Identification |
| CD141 | Special surface markers of cDC1 | Identification |
| CD1c, CD11b | Special surface markers of cDC2 | Identification |
| CD123 | Special surface markers of pDC | Identification |
| CD207, CD1a | Special surface markers of LCs in human (without CD1a of LCs in mice) | Identification |
| CD14, CD1a, CD1c, | Special surface markers of infDCs or Mo-DCs | Identification |
| CD209, CD206, CD303, CD207, CD208 | Other markers of infDCs or Mo-DCs | Identification |
| CD40, CD83, CD80, CD86 | Surface markers of DCs | Co-stimulatory molecules |
| CD40 | Transmembrane protein of DCs | Interaction with T cell or B cell through CD40L-CD40 signaling |
